# Adult expression of Semaphorins and Plexins is essential for motor neuron survival

**DOI:** 10.1038/s41598-023-32943-4

**Published:** 2023-04-11

**Authors:** Aarya Vaikakkara Chithran, Douglas W. Allan, Timothy P. O’Connor

**Affiliations:** 1 Graduate Program in Neuroscience, University of British Columbia, 3402-2215 Wesbrook Mall, Vancouver, BC V6T 1Z3 Canada; 2 Department of Cellular and Physiological Sciences, University of British Columbia, 2350 Health Sciences Mall, Vancouver, BC V6T 1Z3 Canada; 3 Djavad Mowafaghian Centre for Brain Health, University of British Columbia, 2215 Wesbrook Mall, Vancouver, BC V6T 1Z3 Canada

**Keywords:** Neuroscience, Cell death in the nervous system, Cellular neuroscience, Neural circuits

## Abstract

Axon guidance cues direct the growth and steering of neuronal growth cones, thus guiding the axons to their targets during development. Nonetheless, after axons have reached their targets and established functional circuits, many mature neurons continue to express these developmental cues. The role of axon guidance cues in the adult nervous system has not been fully elucidated. Using the expression pattern data available on FlyBase, we found that more than 96% of the guidance genes that are expressed in the *Drosophila melanogaster* embryo continue to be expressed in adults. We utilized the GeneSwitch and TARGET systems to spatiotemporally knockdown the expression of these guidance genes selectively in the adult neurons, once the development was completed. We performed an RNA interference (RNAi) screen against 44 guidance genes in the adult *Drosophila* nervous system and identified 14 genes that are required for adult survival and normal motility. Additionally, we show that adult expression of Semaphorins and Plexins in motor neurons is necessary for neuronal survival, indicating that guidance genes have critical functions in the mature nervous system.

## Introduction

An extraordinary characteristic of the nervous system is the complexity of its wiring. Although there are multiple mechanisms involved in establishing these connections, a key event is the guidance of axons to their specific targets. During the development of the nervous system, axons navigate to their targets in response to various attractive and repulsive guidance cues^1^. Recently, it has become clear that many neurons continue to express developmental guidance genes after functional circuits have been established. Indeed, gene expression studies in the adult rodent brain have identified brain regions that are enriched for genes involved in neuronal development and axon guidance^2^.

The expression of guidance genes in the adult indicates that they likely have additional roles beyond the initial phase of axonal outgrowth and synaptogenesis^3^. For example, blocking the function of Ephrins and class 3 Semaphorins promotes axonal sprouting and regeneration after nerve injury^4–7^. Guidance cues are also known to regulate adult synaptic plasticity. Conditional deletion of the receptor for Netrin-1 (deleted in colorectal cancer/DCC) in adult mice forebrain neurons results in shorter dendritic spines, loss of long-term potentiation, and impaired spatial and recognition memory^8^. Semaphorin 5B has been shown to regulate the elimination of synaptic connections in cultured hippocampal neurons^9^ and Semaphorin 2b- PlexinB signalling was shown to drive homeostatic synaptic plasticity in *Drosophila*^10,11^. Axon guidance genes have also been linked to neurodegenerative diseases. Given their role in axonal guidance and synapse regulation, it has been proposed that changes in guidance protein expression or function may trigger defects in neuronal networks leading to neuronal dysfunction and loss^12^. Thus, based on their known functions during development, their expression in the adult brain and possible implications in neurodegenerative diseases, we examined whether guidance genes are critical for the maintenance and survival of neurons in the mature nervous system.

## Results

### Knockdown of a subset of axon guidance genes reduces adult survival

**Figure 1:**
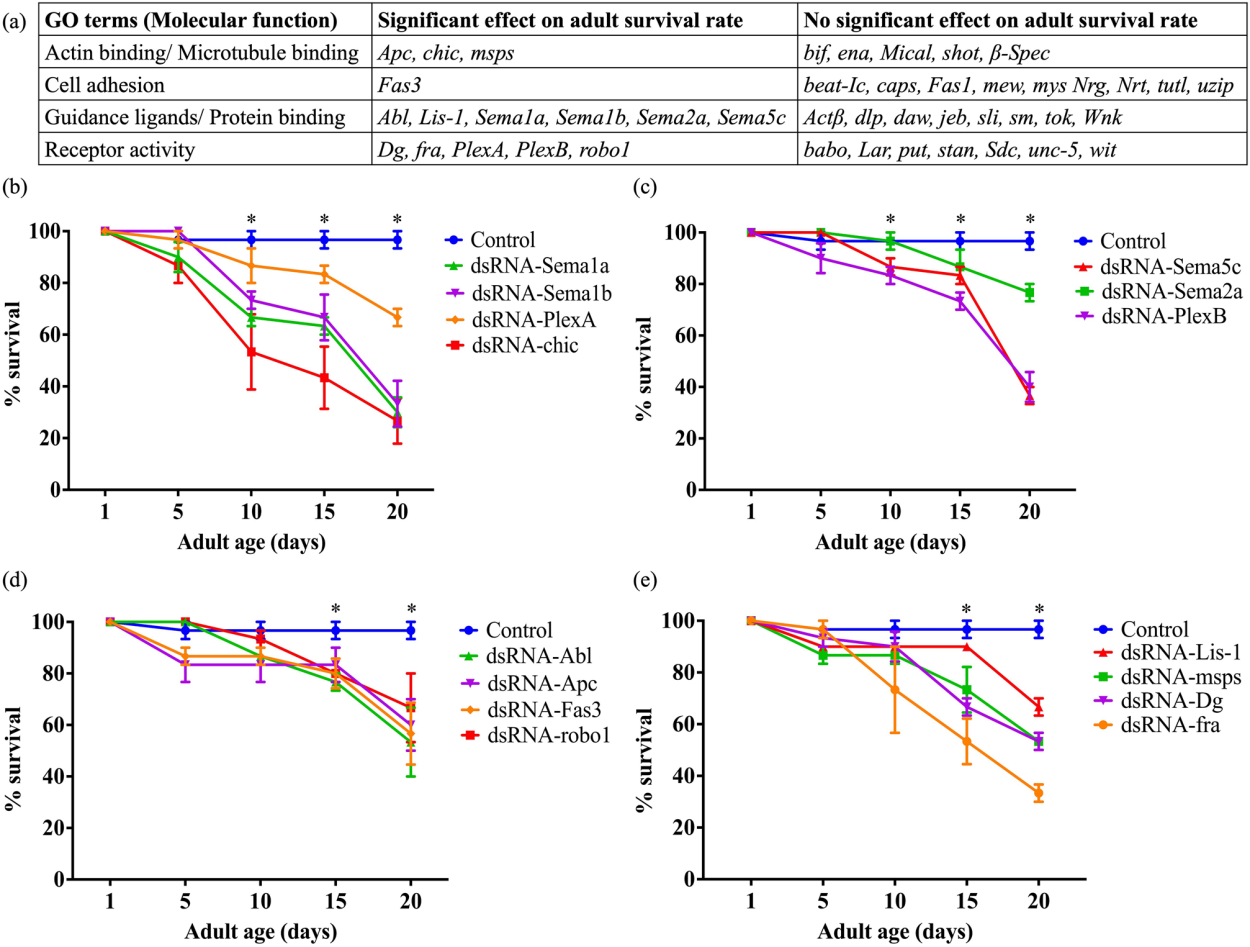
Knockdown of a subset of axon guidance genes reduces adult survival rate. (**a**) A summary of the axon guidance genes that were tested for the impact of knockdown on adult survival rate using the GeneSwitch screen. The axon guidance cues are categorized into four groups based on the molecular function GO terms. (**b**, **c**) Survival analysis curves of axon guidance genes that showed a significant effect on survival from day 9 post eclosion. We note that Sema2a is not significantly different from the control on day 10 (c), however we included these flies in the graph to group the analysis of the Semaphorin and Plexin genes together. (**d**, **e**) Survival analysis curves of axon guidance genes that showed a significant effect from day 14 post eclosion. Data shown are mean ± SEM (p < 0.05, two-way ANOVA and Tukey’s multiple comparison tests; n = 30). ‘*’ indicates the time points when the survival of knockdowns was significantly different from that of the controls.

Using the expression pattern data^13^ available on FlyBase^14,15^, we compared the expression of axon guidance genes in the embryonic (10–24 h) and adult (1–20 days) *Drosophila melanogaster*. We found that more than 96% of the guidance genes expressed in the embryo continue to be expressed in adults. Query results from the RNA-Seq Expression Profile search tool showed that 162 genes expressed in the adult *Drosophila* nervous system were categorized under the biological process Gene Ontology (GO) term ‘axon guidance’ and 122 genes under ‘dendrite morphogenesis’ (Supplementary Tables S1, S2). After excluding transcription factors, 44 genes were prioritized based on their previously known roles in axonal guidance and the availability of genetic tools (Supplementary Tables S3, S4). We refer to these genes collectively as ‘axon guidance genes’. Based on the molecular function GO terms provided on FlyBase, these 44 axon guidance genes were categorized as ‘actin/ microtubule-binding’, ‘cell adhesion’, ‘guidance ligand/ protein binding’ and ‘receptor activity’ (Fig. 1a).

**Figure 2:**
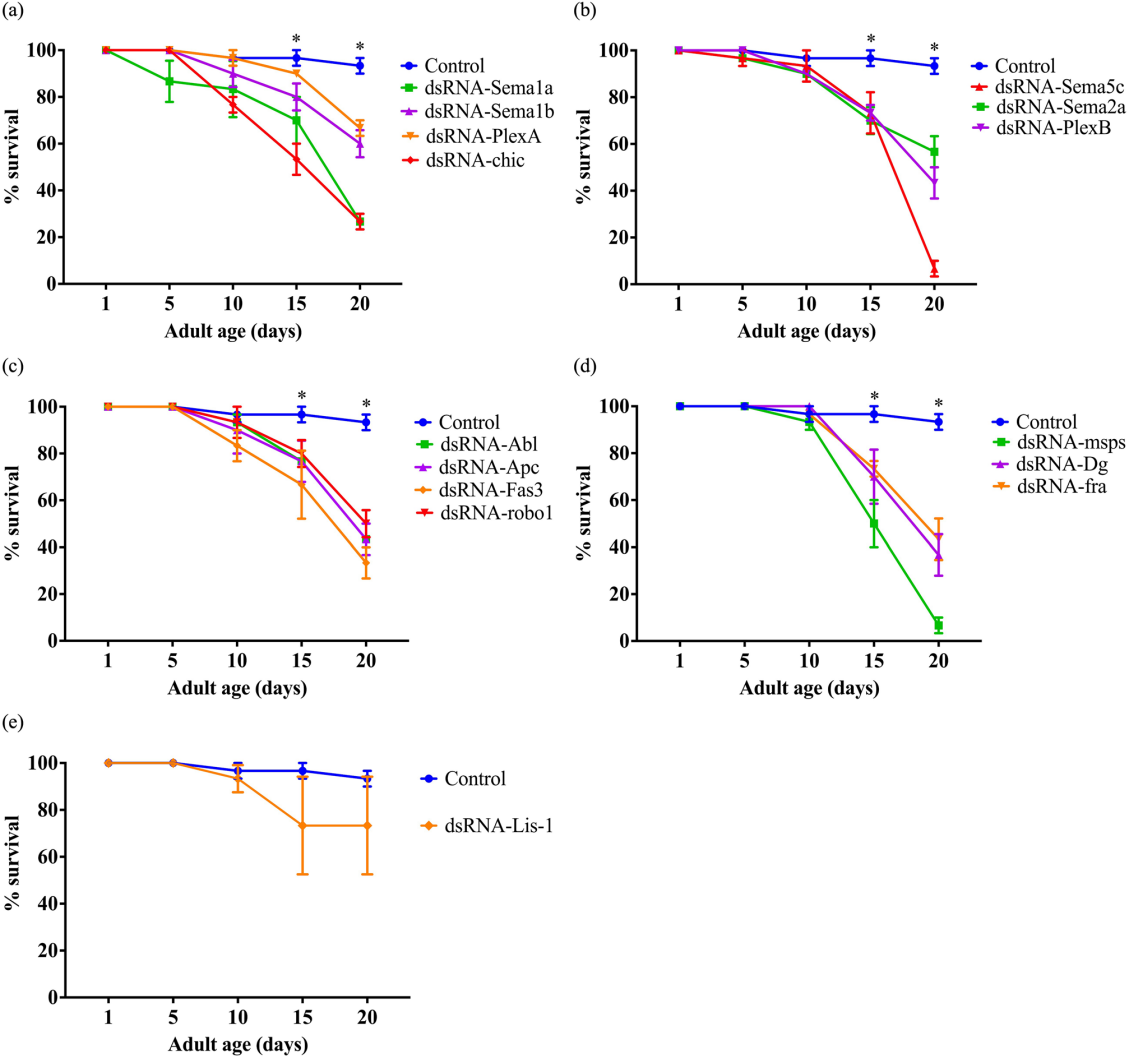
The TARGET system screen confirmed a reduced adult survival rate for 14 axon guidance genes knocked down. (**a**–**d**) Knockdown of *Sema1a, Sema1b, PlexA, chic, Sema5c, Sema2a, PlexB, Abl, Apc, Fas3, robo1, msps, Dg* and *fra* exhibited a significant reduction in the adult survival rate. (**e**) Knockdown of *Lis-*1 did not show a significant effect on the adult survival rate in the TARGET screen*.* Data shown are mean ± SEM (p < 0.05, two-way ANOVA and Tukey’s multiple comparison tests; n = 30). ‘*’ indicates the time points when the survival of knockdowns was significantly different from that of the controls.

In an initial screen to identify whether any genes were essential for viability, we used the GeneSwitch (*elav-GeneSwitch-Gal4*) and TARGET (*elav-Gal4* with *tub-Gal80*^*ts*^) systems^16^ (see Supplementary Table S5) to knockdown the expression of all 44 genes (using multiple *dsRN*A and *shRNA* lines; see Supplementary Table S6) selectively in the adult neurons. Since all the flies used in this study have *UAS-Dicer2* on the X chromosome and since *Drosophila* males have a hyperactive X chromosome, we picked male flies for all the experiments. The hyperactivation of a single X chromosome in male flies increases the transcription of genes to match that of both X chromosomes in females^17,18^. This dosage compensation in *Drosophila* males is due to the increased acetylation at the K16 residue of histone H4 which, in this case, causes transcriptional activation and upregulation of the *UAS-Dicer2* on the X chromosome which in turn enhances the potency of RNAi^19^. In the GeneSwitch screen, the adult-specific knockdown of 15 of the 44 genes showed a significant reduction in the adult survival rate (Fig. 1, Supplementary Fig. S1). The TARGET system screen confirmed these data for 14 of the 15 genes (Fig. 2). For each gene, we used multiple *UAS*-*dsRNA* and -*shRNA* lines as listed in Supplementary Table S6. In Figs. 1, 2 and Supplementary Fig. S1, we report survival data from only the most effective lines. For each *UAS-dsRNA* and* -shRNA* line, we employed two controls- an injection strain control and a *UAS-dsRNA-GFP* control. All controls used in this study are listed in Supplementary Table S7. Since all the controls exhibited similar survival rates in the adults, we report the data from only one control in each survival analysis curve. In the control flies, 97% survive to day 20 post eclosion, whereas the percentage survival post knockdown for the 14 genes was significantly reduced to 27% (*chic*), 30% (*Sema1a*), 33% (*fra, Sema1b*), 37% (*Sema5c*), 40% (*PlexB*), 53% (*Abl, Dg, msps*), 57% (*Fas3*), 60% (*Apc*), 67% (*PlexA, robo1*), and 77% (*Sema2a*) (Fig. 1b–e).

### Motility is impacted by the knockdown of specific axon guidance genes

**Figure 3:**
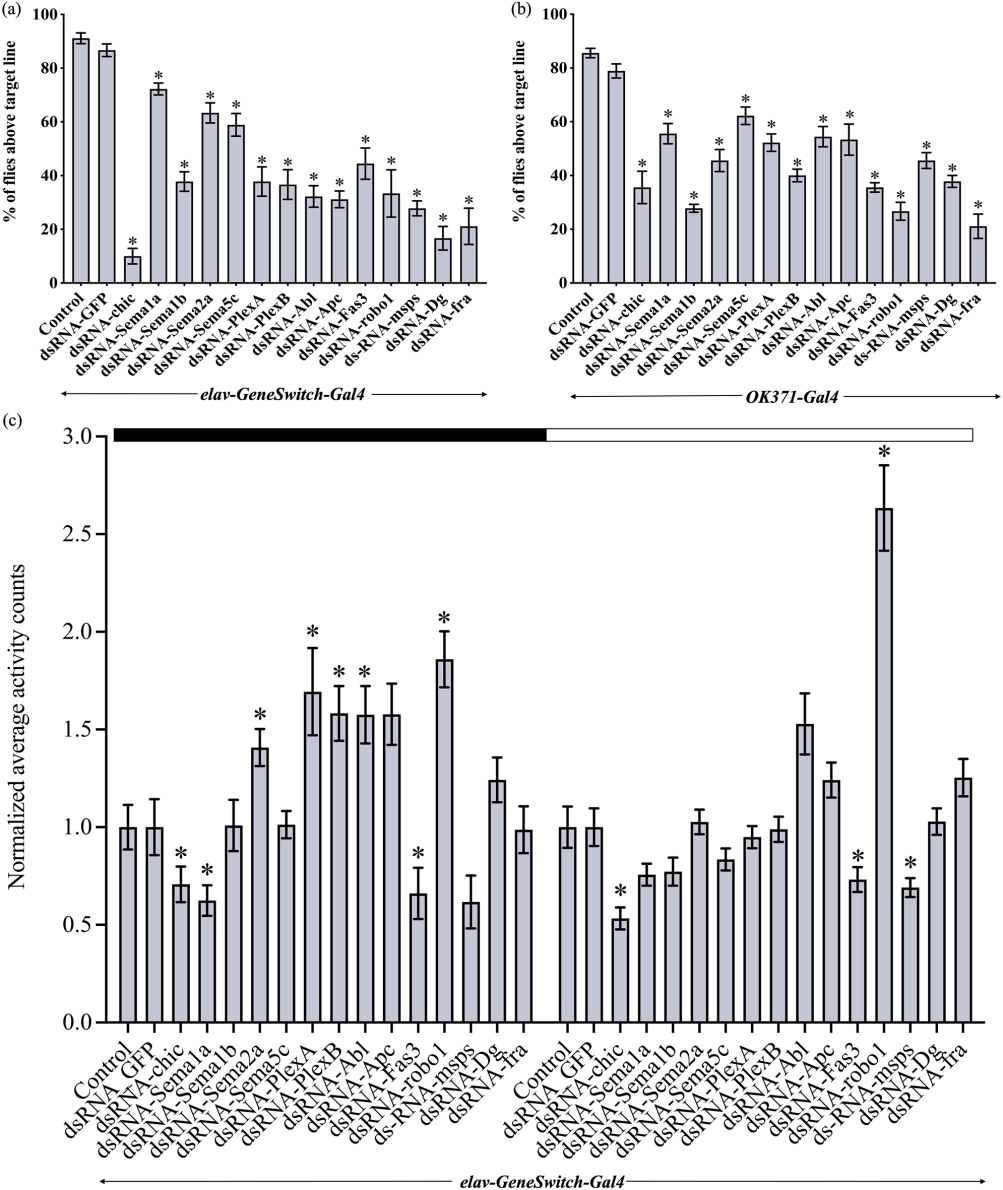
Motility is impacted by the knockdown of specific axon guidance genes (**a**) Effect of pan-neuronal knockdown of axon guidance genes (using *elav*-*GeneSwitch-Gal4*) on adult climbing behaviour. A significant decrease in adult climbing ability was observed after the knockdown. (**b**) Similar observations were made when each gene was knocked down specifically in glutamatergic motor neurons using the *OK371-Gal4*. Data shown are mean ± SEM (‘*’ denotes a significant difference from the control and dsRNA-GFP; p < 0.05, one-way ANOVA and Tukey’s multiple comparison tests; n = 90). (**c**) Effect of pan-neuronal knockdown of axon guidance genes (using *elav*-*GeneSwitch-Gal4*) on adult activity (measured using DAM). The bar graph compares the normalized average activity counts per 12 h for each gene from multiple flies over a period of 3 days in 12 h light and 12 h dark cycle. The black portion (0–12 h) represents the dark cycle, and the white portion (12–24 h) represents the light cycle. Data shown are mean ± SEM (‘*’ denotes a significant difference from the control and dsRNA-GFP; p < 0.05, one-way ANOVA and Tukey’s multiple comparison tests; n = 32).

To examine adult fly behaviour in the period before their survival declines, we focused on these 14 genes and divided them into two groups based on their survival analysis graphs: (1) those that started to show a reduction in adult survival from approximately day 9 post eclosion (*Sema1a, Sema1b, Sema2a, Sema5c, PlexA, PlexB, chic*), and (2) those that started to show a reduction from approximately day 14 post eclosion (*Abl, Apc, Dg, Fas3, fra, msps, robo1*). Using these two time points, we conducted motility assays with group one at 9 days and group two at 14 days, after the initiation of *shRNA* and *dsRNA* expression. Here, we used the *UAS-dsRNA* and *-shRNA* lines that were most effective in the survival assays and employed two controls (an injection strain control and a *UAS-dsRNA-GFP* control) for each line. There was no significant difference between the controls on day 9 and day 14. As an initial examination of motility, we tested whether pan-neuronal knockdown (*elav-GeneSwitch-Gal4*) of the genes impacted adult climbing behaviour. We found that flies that had axon guidance genes knocked down showed a significant climbing defect. Flies that failed to reach the target line were either very slow or did not start climbing even after tapping the vials. In the control flies, the success of climbing ranged from 87 to 94%. We observed the following climbing success rates in knockdowns of *Sema1a* (72%), *Sema2a* (63%), *PlexA* (38%) and *PlexB* (37%). The climbing success rates ranged from 10 to 59% in the knockdowns of the other 10 genes (Fig. 3a).

Since a significant decline in adult climbing behaviour was observed after pan-neuronal knockdown, we tested whether knocking down genes primarily in motor neurons would induce a similar phenotype using a glutamatergic neuron-specific Gal4 driver (*OK371-Gal4*). Again, flies with knocked-down guidance genes showed defects in adult climbing behaviour (Fig. 3b). Comparing the severity of climbing defects caused by the knockdown of each gene using the two drivers, only *chic* and *Apc* showed less severity following the knockdown in motor neurons compared to the pan-neuronal knockdown. The remaining 12 genes showed no significant difference in the severity of the climbing phenotype between the two Gal4 drivers (Fig. 3a, b).

We further examined the motility of adult flies using a *Drosophila* Activity Monitor (DAM) by continuously monitoring individual flies for 3 days. *Fas3* and *chic* knockdown flies showed significantly lower activity counts than the controls throughout the day (both light and dark cycles). Whereas *robo1* knockdown flies exhibited significantly higher activity counts in both cycles. During the dark cycle, the *Sema1a* knockdown flies exhibited significantly lower activity counts whereas *Sema2a*, *PlexA* and *PlexB* knockdown flies exhibited significantly higher activity counts. Flies also showed aberrant activity counts following the knockdown of *Abl and msps* (Fig. 3c).

### Knockdown of Semaphorins and Plexins results in motor neuron death

**Figure 4:**
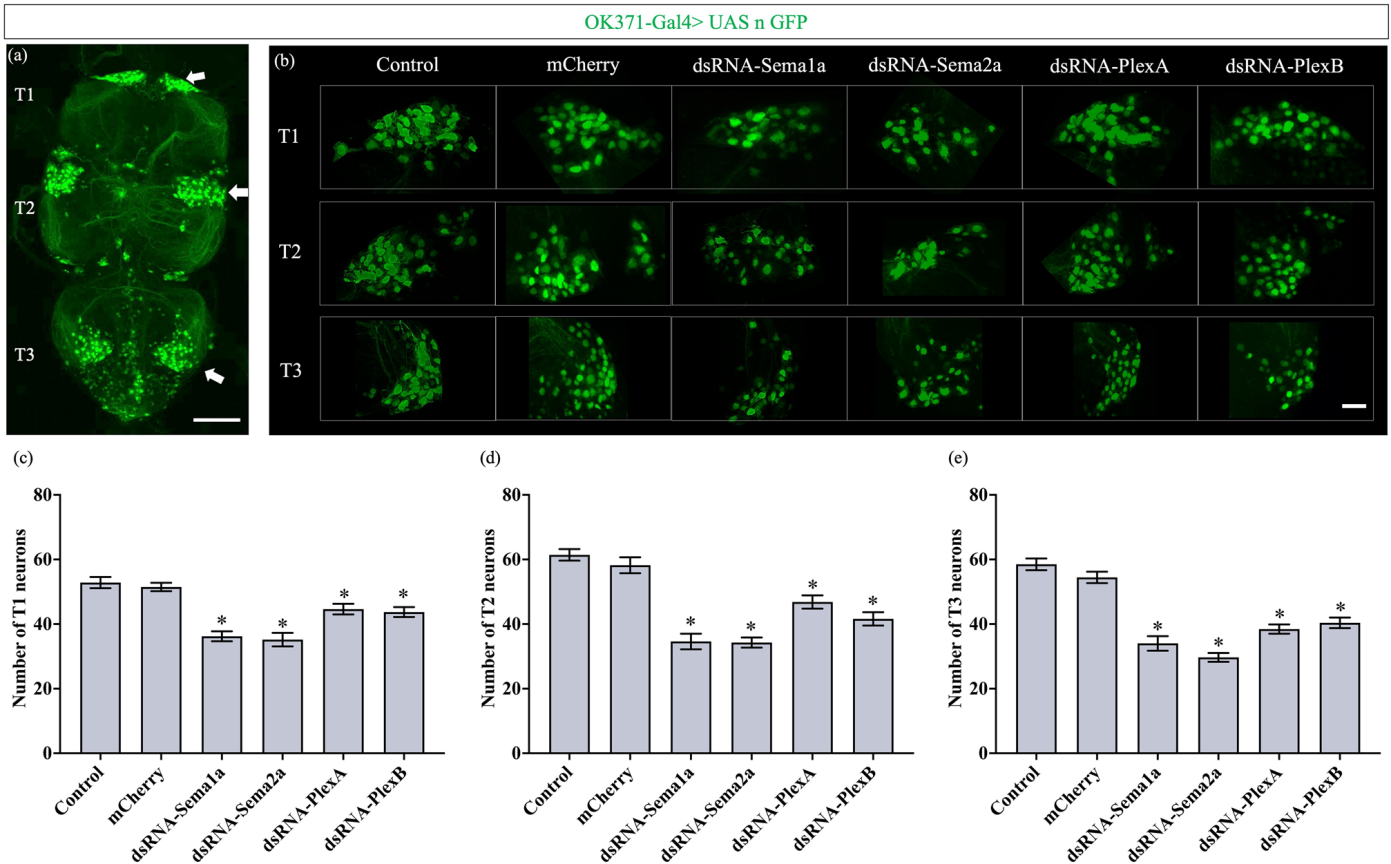
Knockdown of Semaphorins and Plexins results in motor neuron death. (**a**) Adult ventral nerve cord showing neuronal GFP expression driven by *OK371-Gal4*. White arrows point to the location of leg motor neuron cell bodies in T1, T2, and T3 thoracic segments. The scale bar represents 100 µm. (**b**) Effect of glutamatergic knockdown of Semaphorins and Plexins (using *OK371-Gal4*) on adult leg motor neuron survival. A decrease in the number of leg motor neurons was observed after the knockdown of Semaphorins and Plexins genes. The scale bar represents 20 µm. (**c**–**e**) Quantification of adult leg motor neurons in T1, T2, and T3 respectively, following 9 days of axon guidance gene knockdown. Data shown are mean ± SEM (‘*’ denotes a significant difference from the control and mCherry; p < 0.05, one-way ANOVA and Tukey’s multiple comparison tests; n = 20).

To investigate why motility was impacted after knockdown, we examined the number of leg motor neurons in the thoracic clusters of adult ventral nerve cords in a subset of the 14 genes using the *OK371-Gal4* driver. Knockdown of *Sema1a, Sema2a, PlexA* and *PlexB* resulted in a significant loss of motor neurons in each thoracic cluster (T1, T2, T3) (Fig. 4). In T1, total neurons were reduced by 32%, 33%, 30% and 38% in *Sema1a, Sema2a, PlexA* and *PlexB* knockdowns respectively. The following reductions were observed in T2 and T3 segments respectively: 44% and 42% (*Sema1a*), 41% and 49% (*Sema2a*), 32% and 36% (*PlexA*) and 30% and 34% (*PlexB*) (Fig. 4c–e). The number of motor neurons in all knockdowns was significantly lower than their injection strain control and the *mCherry*-control. This was repeated using additional *UAS-dsRNA* lines and controls which corroborated the reduction in leg motor neuron numbers (Supplementary Fig. S2).

## Discussion

Among the 44 axon guidance genes screened, we identified 14 genes (*chic, Sema1a, Sema1b, Sema2a, Sema5c, PlexA, PlexB, Abl, Apc, Fas3, robo1, msps, Dg, fra)* that are essential for adult survival. Mutations in *chic*^20–30^*, Sema1a*^19,24,31^*, Sema2a*^23,24,32^*, Abl*^24,31,33–38^*, Apc*^39^*, Fas3*^27,40^*, robo1*^41,42^*, msps*^27,43,44^*, Dg*^45,46^*,* and *fra*^47^ were previously known to cause lethality during development. This implies that many axon guidance genes are essential throughout the lifespan of *Drosophila*, from development through adulthood. Since we used the GeneSwitch and TARGET systems to restrict the knockdown to adult flies, the observed phenotypes are not a result of developmental defects but are due to defects in the adult nervous system. One important consideration while using RNAi is that it could produce off-target effects where multiple gene expressions will be altered. To address this issue, we picked *UAS-dsRNA* and *-shRNA* lines that were used in previous studies where no off-target effects were reported^48^. In addition, we used multiple *UAS-dsRNA* and *-shRNA* lines to confirm each of the observed phenotypes. It is important to note that *UAS-dsRNA* and *-shRNA* lines that use the phiC31a/AttP site-specific integration system to insert the transgene in a well-characterized location on the chromosome ensure high levels of expression and an insertion in a position that does not affect other genes^49^. We have employed the use of such lines in most of the experiments performed.

The reduction in the survival rate could result from the dysfunction of any number of neuronal populations in the adult nervous system. It is presumed that the functions of the affected neurons are compromised before the flies die. To narrow down the range of neuron populations that could underlie this premature death and to better understand the cellular impact following guidance gene knockdown, we used motility assays and focussed our examination on motor neural circuits. Employing a climbing assay and the DAM system, we found a significant impact on motility in knockdown flies. Defective climbing behavior was observed when the knockdown was mediated either pan-neuronally (using *elav-GeneSwitch-Gal4)* or restricted to motor neurons (using *OK371-Gal4).* One potential limitation of using *OK371-Gal4* is that although it drives expression predominantly in the motor neurons in the adult ventral nerve cord it is also expressed in a few glutamatergic interneurons in the brain^50^. As a result, it is unclear whether axon guidance gene knockdown in non-motor glutamatergic neurons would contribute to the defective climbing behavior. Further analysis of these additional neuronal populations may be required to understand this better. It is also interesting that while axon guidance gene knockdown always resulted in a decreased climbing behavior, the same resulted in either an increased or decreased activity using the DAM system. This may be due to the fact that both of these experiments measure different kinds of motility behaviors. The climbing assay measures the negative geotaxis behavior in a short span (8 s) whereas the DAM system measures locomotion as reflected by the ‘activity counts’ over an extended period of time (3 days on a 12 h dark/12 h light cycle). Defects in the circadian rhythm and sleep may also contribute to the differences in the activity observed using DAM. Since it is beyond the scope of our current study, we did not examine the neural circuits underlying the circadian rhythm.

As pointed out earlier, since the knockdown was restricted to adult flies, these locomotor phenotypes are not the result of developmental defects but are likely due to defects in the adult motor neuron circuit. This was confirmed when the knockdown of *Sema1a, Sema2a, PlexA* and *PlexB* resulted in a significant loss of adult motor neurons, suggesting a role for these guidance cues in maintaining neuronal survival during adulthood. The loss of motor neurons could explain the motility defects demonstrated in the behavioural assays. It is noteworthy that while the adult-specific knockdown of Semaphorins and Plexins resulted in a similar loss of motor neurons, their effects on locomotor activity appeared quite different. Knockdown of *Sema1a* decreased the average activity counts, whereas that of *Sema2a*, *PlexA* and *PlexB* increased the average activity counts. This may be due to the differential effect of knockdown on the neuronal populations controlling circadian rhythm and sleep. Further analysis on the inactive time duration/ sleep of knockdown flies and examination of the neural circuits underlying the circadian rhythm may be required to fully understand this phenomenon.

Although the neuroprotective function of Semaphorins has been previously demonstrated in human^51^ and mouse spinal motor neurons^52^, cultured cerebellar granule cells^53^, and non-neuronal cells^54–57^ during development, our study is the first to show that adult-specific expression of Semaphorins and Plexins is essential for maintaining motor neuron survival in the mature nervous system. In addition to promoting cell survival, Semaphorins and Plexins are also known to evoke responses such as cell proliferation and differentiation^58^. An alternative interpretation of the loss of adult motor neurons is that the lack of Semaphorin-Plexin signalling may be inhibiting proliferation and differentiation into motor neurons. This possibility was ruled out since there is no evidence for adult neurogenesis in the *Drosophila* motor neuron circuits.

Semaphorin/Plexin signalling has been studied throughout the development of embryonic and post-embryonic motor neurons^59–61^. At the *Drosophila* larval neuromuscular junctions, *Sema2b*- *PlexB* signalling has been demonstrated to control presynaptic neurotransmitter release to drive homeostatic plasticity^10,11^. In addition, previous work has shown that *Sema1a, Sema2a, PlexA* and *PlexB* play a range of roles in establishing the circuitry of adult leg motor neurons in *Drosophila*^62^. Here, we show that these very same proteins are essential for maintaining motor neuron survival during adulthood. Thus, Semaphorins and Plexins are important equally for the formation as well as the maintenance of adult neural circuits. Semaphorins and Plexins may act cell autonomously on motor neurons or non-cell autonomously on neighbouring motor neurons to maintain their survival. Further studies on Semaphorin/ Plexin signalling downstream events will be required to better understand the mechanism underlying this phenotype.

The main goal of our study was to examine whether axon guidance genes have an important function in the adult. We identified 14 axon guidance genes that are essential for survival and normal motility. From our database search, we found that many of the embryonic axon guidance genes continue to be expressed in the adult. Gene expression studies in adult mouse brains suggest that although the expression patterns of many genes change dramatically during development, the brain retains a degree of embryological gene expression that is important for the maintenance of established units in the adult brain^63^. Similarly, studies in rhesus monkeys (*Macaca mulatta*) have identified sets of genes that are important equally for the formation as well as the maintenance and plasticity of connections in the adult thalamus^64^. These imply that the expression of axon guidance genes in the adult is not just a “residue” of the developmental pattern that reflects processes occurring when connectivity is established, but is functionally relevant with respect to maintaining the connectivity. Indeed, our results show that the expression of axon guidance genes in adulthood is required for organism viability and neuronal survival.

## Methods

### Prioritizing axon guidance genes for the screen

The high throughput expression pattern data^11^ accessed through the RNA-Seq Expression Profile search tool from FlyBase^12,13^ (www.flybase.org) version FB2013_05 (released September 2013) was used to search for genes expressed in *Drosophila melanogaster* in the embryo (stage-wise expression, 10–24 h) and in the adult (tissue-wise expression in head, 1–20 days post eclosion). An ‘expression on’ filter was used to search for genes expressed above a certain expression profile threshold, namely very low (1–3 RPKM), low (4–10 RPKM), moderate (11–25 RPKM), moderately high (26–50 RPKM), high (51–100 RPKM), very high (101–1000 RPKM) and extremely high (> 1000 RPKM). The resulting dataset was further refined using biological process GO terms ‘axon guidance’ and ‘dendrite morphogenesis’. Transcription factors were excluded using the cellular component GO term ‘nucleus’. Gene list Venn diagram (http://genevenn.sourceforge.net/) was used to categorize the genes into discrete expression levels, ranging from very low to extremely high, and to compare the genes expressed in the embryo and adult *Drosophila*. These data combined with their previously known roles in receptor activity and signalling during neuronal pathfinding, and the availability of genetic tools were used to prioritize genes.

### Fly genetics

Reducing axon guidance gene expression by RNAi in the adult nervous system was carried out using two well-characterized systems: (1) GeneSwitch, (2) TARGET. Gal4 driver line virgin females (see Supplementary Table S5) were crossed with *UAS-dsRNA/ shRNA* males (see Supplementary Table S6) or the appropriate negative control line males (see Supplementary Table S7). In case of the GeneSwitch screen (using *elav-GeneSwitch-Gal4*), crosses were set up at 25 °C. The progeny (F1) was fed with food containing 6.5 µg/mL mifepristone (RU486) from day 1 post eclosion to activate the Gal4-UAS system. In the case of the TARGET screen (using *elav-Gal4 OR OK371-Gal4* with *tub-Gal80*^*ts*^), crosses were set up at 18 °C and the progeny (F1) was switched to 29 °C immediately after eclosion to repress Gal80^ts^ and activate the Gal4-UAS system.

### Fly stocks

*Drosophila melanogaster* stocks used in this study were maintained on standard cornmeal food and at 18, 25, or 29 °C in environment rooms set at 70% humidity. The stocks used in this study are listed in Supplementary Tables S5, S6 and S7.

### Survival assay

F1 progeny males were collected immediately after eclosion. They were maintained in vials of 10 and their survival was recorded every day for the next 20 days. Flies were flipped into fresh vials every week. Survival analysis for the GeneSwitch screen was carried out at 25 °C and that for the TARGET screen was carried out at 29 °C. In both cases, multiple *UAS-dsRNA/ shRNA* lines were tested for each axon guidance gene. For each line tested, 3 vials containing 10 flies were assayed. The results were analyzed using two-way ANOVA and Tukey’s multiple comparison tests using GraphPad Prism.

### Climbing assay

To identify adult climbing defects, F1 male flies were collected immediately after eclosion, maintained in groups of 10 flies per vial and assayed at the desired age. During the climbing assay, flies were transferred to a clean, empty vial with a line drawn 7.5 cm from the bottom. Flies were allowed to acclimatize for 1 min and then tapped to the bottom to induce an innate climbing response. The number of flies that successfully reached the 7.5 cm line in 8 s was recorded. For each genotype, 3 vials were assayed and 3 replicates per vial were performed to ensure an accurate reading for each vial. The results were analyzed using one-way ANOVA and Tukey’s multiple comparison tests using GraphPad Prism.

### *Drosophila* Activity Monitor (DAM) system

The DAM system from TriKinetics was used to monitor the activities of individual flies for 3 days on 12 h light and 12 h dark cycle. F1 male flies were collected immediately after eclosion, maintained in groups of 10 flies per vial and assayed at the desired age at 25 °C. Flies were placed inside the activity monitor tubes for at least 24 h before each experiment to acclimatize to the experimental setup. 32 flies were placed in 5 mm diameter tubes, with one end containing 1 cm worth of food. *Drosophila* activity was recorded every 5 min. The raw data was processed using the actmon R package publicly available online at https://github.com/kazi11/actmon (kindly provided by Jeff Stafford). Data from flies that died during the experiment were discarded. The processed results were analyzed using one-way ANOVA and Tukey’s multiple comparison tests using GraphPad Prism.

### Image acquisition and leg motor neuron quantification

To examine the impact of knockdown at a neuronal level, adult male *Drosophila* ventral nerve cords (VNCs) were dissected in ice cold 1 × PBS, over a period of 30 min. The VNCs were fixed, on poly-Lysine coated slides, in 4% PFA at room temperature for 30 min followed by 3 × 5 min washes with 0.1% PBT. They were mounted in Vectashield mounting media (Vector Laboratories Inc.), coverslipped and sealed with nail-polish. All images were acquired on a 20X objective at a 1.0-fold magnification using the Fast Airyscan feature on a Zeiss LSM 880 inverted confocal microscope. Images were taken in a Z-stack and the leg motor neurons were quantified in a 3D model (using Zen-blue image processing software) separately for the T1, T2 and T3 segments. The results were analysed using one-way ANOVA and Tukey’s multiple comparison tests using GraphPad Prism.

## Supplementary Information

Supplementary Information.

## Data Availability

The datasets generated during and/ or analyzed during the current study are available from the corresponding author on request.
