# Supplementary material for: Adult expression of Semaphorins and Plexins is essential for motor neuron survival
Source: Sci Rep. 2023 Apr 11;13:5894. doi: 10.1038/s41598-023-32943-4 (PMC10090137; doi:10.1038/s41598-023-32943-4)
Supplement: Supplementary file 1 — Supplementary Information. [file 41598_2023_32943_MOESM1_ESM.pdf]

Supplementary Information

Adult expression of Semaphorins and Plexins is essential for motor neuron survival

Aarya Vaikakkara Chithran[1,2], Douglas W. Allan[2,3] and Timothy P. O'Connor*[2,3]
[1] Graduate Program in Neuroscience, 3402-2215 Wesbrook Mall, University of British Columbia, Vancouver BC, Canada V6T 1Z3
[2] Department of Cellular and Physiological Sciences, 2350 Health Sciences Mall, University of British Columbia, Vancouver BC, V6T 1Z3
[3] Djavad Mowafaghian Centre for Brain Health, 2215 Wesbrook Mall, University of British Columbia, Vancouver BC V6T 1Z3
*Email: timothy.oconnor@ubc.ca

Supplementary Table S1. List of 162 genes categorized under the GO term 'axon guidance' that are expressed in the adult *Drosophila* nervous system.

#	Symbol	Name	Annotation ID
1	14-3-3ε	14-3-3ε	CG31196
2	aay	astray	CG3705
3	Abl	Abl tyrosine kinase	CG4032
4	acj6	abnormal chemosensory jump 6	CG9151
5	Acsl	Acyl-CoA synthetase long-chain	CG8732
6	ago	archipelago	CG15010
7	Alk	Alk	CG8250
8	aos	argos	CG4531
9	AP-1σ	Adaptor Protein complex 1, σ subunit	CG5864
10	ap	apterous	CG8376
11	Apc	APC-like	CG1451
12	Apc2	Adenomatous polyposis coli tumor suppressor homolog 2	CG6193
13	babo	baboon	CG8224
14	beat-Ia	beaten path Ia	CG4846
15	beat-Ic	beaten path Ic	CG4838
16	bif	bifocal	CG1822
17	bon	bonus	CG5206
18	brat	brain tumor	CG10719
19	bsk	basket	CG5680
20	bur	burgundy	CG9242
21	CadN	Cadherin-N	CG7100
22	caps	capricious	CG11282

23	Cdc42	Cdc42	CG12530
24	Cdk8	Cyclin-dependent kinase 8	CG10572
25	CG4203	-	CG4203
26	chb	chromosome bows	CG32435
27	Chi	Chip	CG3924
28	chic	chickadee	CG9553
29	CkIIα	casein kinase IIα	CG17520
30	ckn	caskin	CG12424
31	comm	commissureless	CG17943
32	dac	dachshund	CG4952
33	daw	dawdle	CG16987
34	Dbx	Dbx	CG42234
35	Dg	Dystroglycan	CG18250
36	dlp	dally-like	CG32146
37	dnt	doughnut on 2	CG17559
38	dock	dreadlocks	CG3727
39	drl	derailed	CG17348
40	Dscam1	Down syndrome cell adhesion molecule 1	CG17800
41	E(z)	Enhancer of zeste	CG6502
42	egh	egghead	CG9659
43	en	engrailed	CG9015
44	ena	enabled	CG15112
45	Ephrin	Ephrin	CG1862
46	Fas1	Fasciclin 1	CG6588
47	Fas3	Fasciclin 3	CG5803
48	Fmr1	Fmr1	CG6203
49	Fps85D	Fps oncogene analog	CG8874
50	fra	frazzled	CG8581
51	gogo	golden goal	CG32227
52	gt	giant	CG7952
53	gukh	GUK-holder	CG31043
54	Hr51	Hormone receptor 51	CG16801
55	Hrb27C	Heterogeneous nuclear ribonucleoprotein at 27C	CG10377
56	Hsc70-4	Heat shock protein cognate 4	CG4264
57	hts	hu li tai shao	CG43443
58	if	inflated	CG9623
59	InR	Insulin-like receptor	CG18402
60	jeb	jelly belly	CG30040
61	jing	jing	CG9397
62	Khc	Kinesin heavy chain	CG7765
63	kis	kismet	CG3696

64	Klp64D	Kinesin-like protein at 64D	CG10642
65	Kr	Kruppel	CG3340
66	kuz	kuzbanian	CG7147
67	LanA	Laminin A	CG10236
68	Lar	Leukocyte-antigen-related-like	CG10443
69	lea	leak	CG5481
70	Liprin-α	Liprin-α	CG11199
71	lola	longitudinals lacking	CG12052
72	meigo	medial glomeruli	CG5802
73	mew	multiple edematous wings	CG1771
74	Mical	Molecule interacting with CasL	CG33208
75	mid	midline	CG6634
76	msn	misshapen	CG16973
77	msps	mini spindles	CG5000
78	mys	myospheroid	CG1560
79	N	Notch	CG3936
80	Nedd4	Nedd4	CG42279
81	nerfin-1	nervous fingers 1	CG13906
82	NetA	Netrin-A	CG18657
83	NetB	Netrin-B	CG10521
84	Nf-YC	Nuclear factor Y-box C	CG3075
85	NijA	Ninjurin A	CG6449
86	NiPp1	Nuclear inhibitor of Protein phosphatase 1	CG8980
87	not	non-stop	CG4166
88	Nrt	Neurotactin	CG9704
89	NT1	Neurotrophin 1	CG42576
90	nvy	nervy	CG3385
91	otk	off-track	CG8967
92	Pak	PAK-kinase	CG10295
93	pdm3	pou domain motif 3	CG42698
94	Pka-R2	cAMP-dependent protein kinase R2	CG15862
95	plexA	plexin A	CG11081
96	plexB	plexin B	CG17245
97	pod1	pod1	CG4532
98	Pp1-87B	Protein phosphatase 1 at 87B	CG5650
99	pros	prospero	CG17228
100	Psc	Posterior sex combs	CG3886
101	ptc	patched	CG2411
102	Ptp52F	Ptp52F	CG18243
103	Ptp61F	Protein tyrosine phosphatase 61F	CG9181
104	Ptp69D	Protein tyrosine phosphatase 69D	CG10975

105	put	punt	CG7904
106	Rab6	Rab6	CG6601
107	Rac1	Rac1	CG2248
108	raptor	raptor	CG4320
109	ras	raspberry	CG1799
110	retn	retained	CG5403
111	Rheb	Ras homolog enriched in brain ortholog (H. sapiens)	CG1081
112	Rho1	Rho1	CG8416
113	RhoGAP93B	Rho GTPase activating protein at 93B	CG3421
114	RhoGEF64C	Rho guanine nucleotide exchange factor at 64C	CG32239
115	Rich	RIC1 homolog	CG9063
116	robo	roundabout	CG13521
117	robo3	robo3	CG5423
118	run	runt	CG1849
119	S6k	RPS6-p70-protein kinase	CG10539
120	sbb	scribbler	CG5580
121	scb	scab	CG8095
122	Sdc	Syndecan	CG10497
123	sec15	sec15	CG7034
124	Sema-1a	Sema-1a	CG18405
125	Sema-1b	Sema-1b	CG6446
126	Sema-2a	Sema-2a	CG4700
127	Sema-2b	Semaphorin-2b	CG33960
128	Sema-5c	Semaphorin-5c	CG5661
129	sens	senseless	CG32120
130	seq	sequoia	CG32904
131	shg	shotgun	CG3722
132	sim	single-minded	CG7771
133	sli	slit	CG43758
134	sm	smooth	CG9218
135	Smox	Smad on X	CG2262
136	SNF1A	SNF1A/AMP-activated protein kinase	CG3051
137	SoxN	SoxNeuro	CG18024
138	spen	split ends	CG18497
139	spz5	spatzle 5	CG9972
140	Sra-1	specifically Rac1-associated protein 1	CG4931
141	stan	starry night	CG11895
142	Tig	Tiggrin	CG11527
143	tok	tolkin	CG6863
144	Tor	Target of rapamycin	CG5092
145	Trim9	Trim9	CG31721

146	trio	trio	CG18214
147	trx	trithorax	CG8651
148	ttv	tout-velu	CG10117
149	tup	tailup	CG10619
150	tutl	turtle	CG15427
151	unc-5	unc-5	CG8166
152	unc-104	unc-104 ortholog	CG8566
153	Unc-115a	-	CG31352
154	uzip	unzipped	CG3533
155	Vav	Vav ortholog (H. sapiens)	CG7893
156	velo	veloren	CG10107
157	Wnk	WNK homolog	CG7177
158	Wnt5	Wnt oncogene analog 5	CG6407
159	Xe7	Xe7	CG2179
160	XNP	XNP	CG4548
161	β-Spec	β Spectrin	CG5870
162	βTub60D	β-Tubulin at 60D	CG3401

Supplementary Table S2. List of 122 genes categorized under the GO term 'dendrite morphogenesis' that are expressed in the adult *Drosophila* nervous system.

#	Symbol	Name	Annotation ID
1	Aats-gln	Glutaminyl-tRNA synthetase	CG10506
2	Aats-gly	Glycyl-tRNA synthetase	CG6778
3	Aats-trp	Tryptophanyl-tRNA synthetase	CG9735
4	ab	abrupt	CG43860
5	Acf1	ATP-dependent chromatin assembly factor large subunit	CG1966
6	acj6	abnormal chemosensory jump 6	CG9151
7	Actβ	Activin-β	CG11062
8	Adf1	Adh transcription factor 1	CG15845
9	aop	anterior open	CG3166
10	Arc42	Arc42	CG4703
11	Ark	Apaf-1-related-killer	CG6829
12	asf1	anti-silencing factor 1	CG9383
13	babo	baboon	CG8224
14	Bap55	Brahma associated protein 55kD	CG6546
15	Bap60	Brahma associated protein 60kD	CG4303
16	bigmax	bigmax	CG3350
17	bon	bonus	CG5206
18	brm	brahma	CG5942
19	Caf1	Chromatin assembly factor 1 subunit	CG4236
20	Cdc42	Cdc42	CG12530
21	CG2678	-	CG2678
22	CG4328	-	CG4328
23	CG5343	-	CG5343
24	CG9104	-	CG9104
25	CG34340	-	CG34340
26	chinmo	Chronologically inappropriate morphogenesis	CG31666
27	chm	chameau	CG5229
28	ci	cubitus interruptus	CG2125
29	comm	commissureless	CG17943
30	Crg-1	Circadianly Regulated Gene	CG32788
31	ct	cut	CG11387
32	Cul-3	Cullin-3	CG42616
33	d4	d4	CG2682
34	dally	division abnormally delayed	CG4974
35	Dcr-1	Dicer-1	CG4792
36	Dhc64C	Dynein heavy chain 64C	CG7507
37	Dlic	Dynein light intermediate chain	CG1938

38	E(bx)	Enhancer of bithorax	CG32346
39	E(z)	Enhancer of zeste	CG6502
40	E2f	E2F transcription factor	CG6376
41	EcR	Ecdysone receptor	CG1765
42	Elongin-C	Elongin C	CG9291
43	ems	empty spiracles	CG2988
44	ena	enabled	CG15112
45	esc	extra sexcombs	CG14941
46	Fmr1	Fmr1	CG6203
47	fra	frazzled	CG8581
48	fru	fruitless	CG14307
49	futsch	futsch	CG34387
50	Gcn5	Gcn5 ortholog	CG4107
51	gro	groucho	CG8384
52	ham	hamlet	CG31753
53	HmgD	High mobility group protein D	CG17950
54	Iswi	Imitation SWI	CG8625
55	jumu	jumeau	CG4029
56	Khc	Kinesin heavy chain	CG7765
57	kn	knot	CG10197
58	kni	knirps	CG4717
59	l(3)mbt	lethal (3) malignant brain tumor	CG5954
60	Lis-1	Lissencephaly-1	CG8440
61	mbf1	multiprotein bridging factor 1	CG4143
62	MED4	Mediator complex subunit 4	CG8609
63	MED11	Mediator complex subunit 11	CG6884
64	MED24	Mediator complex subunit 24	CG7999
65	MEP-1	-	CG1244
66	Mi-2	-	CG8103
67	Mical	Molecule interacting with CasL	CG33208
68	Nak	Numb-associated kinase	CG10637
69	Nc	Nedd2-like caspase	CG8091
70	Not1	Not1	CG34407
71	Nrg	Neuroglian	CG1634
72	nvy	nervy	CG3385
73	Pi3K92E	Pi3K92E	CG4141
74	prel	preli-like	CG8806
75	pros	prospero	CG17228
76	Ptp69D	Protein tyrosine phosphatase 69D	CG10975
77	Ptx1	Ptx1	CG1447
78	pum	pumilio	CG9755

79	put	punt	CG7904
80	pygo	pygopus	CG11518
81	Rab5	Rab5	CG3664
82	Rac1	Rac1	CG2248
83	Rfx	Rfx	CG6312
84	Rho1	Rho1	CG8416
85	rictor	rapamycin-insensitive companion of Tor	CG8002
86	robl	roadblock	CG10751
87	robo	roundabout	CG13521
88	Rpd3	Rpd3	CG7471
89	run	runt	CG1849
90	S6k	RPS6-p70-protein kinase	CG10539
91	scrt	scratch	CG1130
92	Sema-1a	Sema-1a	CG18405
93	seq	sequoia	CG32904
94	shot	short stop	CG18076
95	shrb	shrub	CG8055
96	Sin1	SAPK-interacting protein 1	CG10105
97	Sin3A	Sin3A	CG8815
98	Sirt2	Sirt2	CG5085
99	sli	slit	CG43758
100	SMC1	SMC1	CG6057
101	Smox	Smad on X	CG2262
102	Snr1	Snf5-related 1	CG1064
103	Sox14	Sox box protein 14	CG3090
104	sqz	squeeze	CG5557
105	stan	starry night	CG11895
106	Su(z)12	Su(z)12	CG8013
107	sv	shaven	CG11049
108	Tab2	TAK1-associated binding protein 2	CG7417
109	Taf4	TBP-associated factor 4	CG5444
110	Tango10	Transport and Golgi organization 10	CG1841
111	TER94	TER94	CG2331
112	tgo	tango	CG11987
113	Tm1	Tropomyosin 1	CG4898
114	Tor	Target of rapamycin	CG5092
115	trc	tricornered	CG8637
116	trh	trachealess	CG42865
117	ttk	tramtrack	CG1856
118	Ube3a	Ubiquitin protein ligase E3A	CG6190
119	usp	ultraspiracle	CG4380

120	vvl	ventral veins lacking	CG10037
121	W	Wrinkled	CG5123
122	wit	wishful thinking	CG10776

Supplementary Table S3. Expression levels of genes categorized under the GO term 'axon guidance' (excluding transcription factors) in the adult *Drosophila* nervous system. Genes in bold text were prioritized for the screen. (RPKM- Reads Per Kilobase of transcript per Million reads mapped)

**Very low (1- 3 RPKM)**	**Low (4-10 RPKM)**	**Moderate (11-25 RPKM)**	**Moderately high (26-50 RPKM)**
**plexin B**	tout-velu	**Leukocyte-antigen-related-like**	astray
doughnut on 2	Laminin A	Kinesin-like protein at 64D	Acyl-CoA synthetase long-chain
**multiple edematous wings**	Netrin-B	Ras homolog enriched in brain ortholog (H. sapiens)	Adaptor Protein complex 1, σ subunit
Ptp52F	brain tumor	**plexin A**	**bifocal**
Netrin-A	Protein tyrosine phosphatase 69D	**capricious**	burgundy
patched	Liprin-α	**APC-like**	**chickadee**
Trim9	**starry night**	**enabled**	**dawdle**
golden goal	caskin	misshapen	egghead
**beaten path Ic**	**roundabout**	trio	**Fasciclin 1**
beaten path Ia	archipelago	**Dystroglycan**	Fmr1
leak	derailed	**Sema-1a**	Heterogeneous nuclear ribonucleoprotein at 27C
**Semaphorin-5c**	Down syndrome cell adhesion molecule 1	**jelly belly**	Kinesin heavy chain
sec15	commissureless	Unc-115a	**myospheroid**
scab	Insulin-like receptor	**Molecule interacting with CasL**	PAK-kinase
**frazzled**	Ephrin	β-Tubulin at 60D	cAMP-dependent protein kinase R2
spatzle 5	GUK-holder	Rho GTPase activating protein at 93B	Protein phosphatase 1 at 87B
	**dally-like**	dreadlocks	Rac1
	Rho guanine nucleotide exchange factor at 64C	**Abl tyrosine kinase**	raspberry
	Semaphorin-2b	hu li tai shao	Rho1
	**shotgun**	**slit**	RPS6-p70-protein kinase
	CG4203	pod1	**Syndecan**
	raptor	**Sema-2a**	Smad on X
	argos	**mini spindles**	Tiggrin
	specifically Rac1-associated protein 1	**Fasciclin 3**	**tolkin**
	Target of rapamycin	**Sema-1b**	**turtle**
	robo3	Rab6	unc-104 ortholog

	medial glomeruli	**WNK homolog**	**unzipped**
	Wnt oncogene analog 5	**baboon**	Xe7
	Ninjurin A	Alk	**β Spectrin**
	Cadherin-N	Fps oncogene analog	
	kuzbanian	**smooth**	
	Vav ortholog (H. sapiens)		
	**punt**		
	**unc-5**		
	RIC1 homolog		
	inflated		
	**Neurotactin**		

Supplementary Table S4. Expression levels of genes categorized under the GO term 'dendrite morphogenesis' (excluding transcription factors) in the adult *Drosophila* nervous system. Genes in bold text were prioritized for the screen. (RPKM- Reads Per Kilobase of transcript per Million reads mapped)

**Very low (1- 3 RPKM)**	**Low (4-10 RPKM)**
Wrinkled	Glutaminyl-tRNA synthetase
Nedd2-like caspase	Glycyl-tRNA synthetase
**frazzled**	Tryptophanyl-tRNA synthetase
	**Activin-β**
	Arc42
	Apaf-1-related-killer
	anti-silencing factor 1
	**baboon**
	Brahma associated protein 55kD
	CG9104
	Chronologically inappropriate morphogenesis
	commissureless
	Cullin-3
	division abnormally delayed
	Dynein heavy chain 64C
	Dynein light intermediate chain
	Enhancer of bithorax
	Elongin C
	**enabled**
	Fmr1
	futsch
	High mobility group protein D
	Kinesin heavy chain
	**Lissencephaly-1**
	**Molecule interacting with CasL**
	Numb-associated kinase
	Not1
	**Neuroglian**
	Pi3K92E
	preli-like
	Protein tyrosine phosphatase 69D
	pumilio
	**punt**
	Rab5
	Rac1
	Rho1
	rapamycin-insensitive companion of Tor

	roadblock
	**roundabout**
	RPS6-p70-protein kinase
	**Sema-1a**
	**short stop**
	shrub
	SAPK-interacting protein 1
	Sirt2
	**slit**
	Smad on X
	**starry night**
	Transport and Golgi organization 10
	TER94
	Tropomyosin 1
	Target of rapamycin
	**wishful thinking**

Supplementary Table S5. List of Gal4 driver lines used in the study

Assay	Driver	Comments
Survival assay	*elav-Gal4*^{c155}*, UAS-Dicer2; tubGal80*^{ts}*, UAS-nGFP*	Pan-neuronal knockdown (TARGET)
Survival assay	*elav-Gal4*^{c155}*, UAS-Dicer2; ; tubGal80*^{ts}*, UAS-nGFP*	Pan-neuronal knockdown (TARGET)
Survival assay + climbing assay + DAM	*UAS-Dicer2; ; elavGS, UAS-nGFP*	Pan-neuronal knockdown (GeneSwitch)
Climbing assay + Leg motoneuron analysis	*UAS-Dicer2; OK371-Gal4; tubGal80*^{ts}*, UAS-nGFP*	Glutamatergic knockdown (TARGET)

Supplementary Table S6. List of *UAS-dsRNA* and *-shRNA* lines used in the study

Drosophila gene	Mammalian homolog	Library	Stock#	Genotype	Target[48]
Abl	*Abl*	BDSC	28325	y^1 v^1; P{TRiP.JF02960}attP2	data N/A
Actβ	*Actβ-A/B*	BDSC	42493	y^1 v^1; P{TRiP.HMJ02057}attP40	all isoforms
		BDSC	29597	y^1 v^1; P{TRiP.JF03276}attP2	all isoforms
		VDRC	108663	P{KK101617}VIE-260B	all isoforms
Apc	*Apc*	BDSC	28582	y^1 v^1; P{TRiP.HM05070}attP2	all isoforms
		BDSC	34869	y^1 sc* v^1; P{TRiP.HMS00188}attP2	all isoforms
babo	*Tgfbr1*	BDSC	40866	y^1 v^1; P{TRiP.HMS02033}attP40	all isoforms
		BDSC	25933	y^1 v^1; P{TRiP.JF01953}attP2	all isoforms
		VDRC	106092	P{KK108186}VIE-260B	all isoforms
beat-Ic	-	VDRC	105066	P{KK113293}VIE-260B	all isoforms
bif	-	BDSC	28372	y^1 v^1; P{TRiP.JF03009}attP2	all isoforms
		VDRC	109722	P{KK105557}VIE-260B	all isoforms
cac	*Cacna1-a/b*	BDSC	27244	y^1 v^1; P{TRiP.JF02572}attP2	all isoforms
caps	*Lrrn2*	BDSC	28020	y^1 v^1; P{TRiP.JF02854}attP2	all isoforms
chic	*Pfn4*	BDSC	34523	y^1 sc^* v^1; P{TRiP.HMS00550}attP2	all isoforms
		VDRC	102759	P{KK112358}VIE-260B	all isoforms
dlp	*Gpc4*	BDSC	34089	y^1 sc* v^1; P{TRiP.HMS00875}attP2	all isoforms
		BDSC	34091	y^1 sc* v^1; P{TRiP.HMS00903}attP2	all isoforms
daw	*Actβ-C*	BDSC	50911	y^1 v^1; P{TRiP.HMJ03135}attP40	all isoforms
		BDSC	34974	y^1 sc^* v^1; P{TRiP.HMS01110}attP2	all isoforms
		VDRC	105309	P{KK110248}VIE-260B	all isoforms
Dg	*Dag1*	BDSC	34895	y^1 sc* v^1; P{TRiP.HMS01240}attP2	all isoforms
		VDRC	107029	P{KK100828}VIE-260B	all isoforms
ena	*Enah/ Evl*	BDSC	39034	y^1 sc^* v^1; P{TRiP.HMS01953}attP2	all isoforms
		BDSC	31582	y^1 v^1; P{TRiP.JF01155}attP2	all isoforms
Fas1	-	BDSC	42887	y^1 sc* v^1; P{TRiP.HMS02580}attP40	all isoforms
		VDRC	23014	w^{1118}; P{GD12817}v23014	all isoforms
		VDRC	23015	w^{1118}; P{GD12817}v23015	all isoforms
Fas3	-	VDRC	939	w^{1118}; P{GD80}v939	all isoforms
		VDRC	940	w^{1118}; P{GD80}v940	all isoforms
		VDRC	3091	w^{1118}; P{GD2576}v3091	all isoforms
		VDRC	26850	w^{1118}; P{GD13161}v26850	1 out of 7 isoforms (Fas3-RC)
fra	*Dcc*	BDSC	31469	y^1 v^1; P{TRiP.JF01231}attP2	all isoforms
		BDSC	31664	y^1 v^1; P{TRiP.JF01457}attP2	all isoforms
		BDSC	40826	y^1 sc* v^1; P{TRiP.HMS01147}attP2	all isoforms
		VDRC	29910	w^{1118}; P{GD14401}v29910	all isoforms
		VDRC	29909	w^{1118}; P{GD14401}v29909/TM3	all isoforms
jeb	-	VDRC	103047	P{KK111857}VIE-260B	all isoforms

Lar	*Ptpr-d/ f*	BDSC	40938	y^1 v^1; P{TRiP.HMS02186}attP40	all isoforms
		BDSC	34965	y^1 sc* v^1; P{TRiP.HMS00822}attP2	all isoforms
		VDRC	107996	P{KK100581}VIE-260B	all isoforms
Lis-1	*Lis-1*	BDSC	35043	y^1 sc^* v^1; P{TRiP.HMS01457}attP2	3 out of 4 isoforms (Lis-1-RA, RB, RF)
		BDSC	28663	y^1 v^1; P{TRiP.JF03078}attP2	all isoforms
msps	*Ckap5*	BDSC	38990	y^1 sc^* v^1; P{TRiP.HMS01906}attP40/CyO	all isoforms
		BDSC	31138	y^1 v^1; P{TRiP.JF01613}attP2	all isoforms
Mical	*Mical-2/3*	BDSC	31148	y^1 v^1; P{TRiP.JF01625}attP2	all isoforms
		VDRC	105837	P{KK102751}VIE-260B	all isoforms
mew	*Itga-6/7*	BDSC	27543	y^1 v^1; P{TRiP.JF02694}attP2	all isoforms
		BDSC	44553	y^1 sc* v^1; P{TRiP.HMS02849}attP2	all isoforms
		VDRC	44890	w^{1118}; P{GD1230}v44890	all isoforms
mys	*Itgb-1/2*	BDSC	33642	y^1 v^1; P{TRiP.HMS00043}attP2	all isoforms
		VDRC	29620	w^{1118}; P{GD15002}v29620/CyO; MKRS/ TM6B, Tb	all isoforms
Nrg	*Nrcam*	BDSC	37496	y^1 sc^* v^1; P{TRiP.HMS01638}attP40	all isoforms
		BDSC	28724	y^1 v^1; P{TRiP.JF03151}attP2	all isoforms
		VDRC	107991	P{KK100482}VIE-260B	all isoforms
		VDRC	6688	w^{1118}; ; P{GD82}v6688	all isoforms
Nrt	-	BDSC	28742	y^1 v^1; P{TRiP.JF03170}attP2	all isoforms
		VDRC	106080	P{KK106657}VIE-260B	all isoforms
PlexA	*Plxna*	BDSC	30483	y^1 sc* v^1; P{TRiP.HM05221}attP2	all isoforms
		VDRC	107004	P{KK101499}VIE-260B	all isoforms
PlexB	*Plxnb*	BDSC	28911	y^1 v^1; P{TRiP.HM05122}attP2	all isoforms
		VDRC	8382	w^{1118}; P{GD2500}v8382	all isoforms
		VDRC	8383	w^{1118}; P{GD2500}v8383	all isoforms
		VDRC	12165	w^{1118}; P{GD3150}v12165	all isoforms
		VDRC	12167	w^{1118}; P{GD3150}v12167	all isoforms
		VDRC	27219	w^{1118}; P{GD14473}v27219	all isoforms
		VDRC	27220	w^{1118}; P{GD14473}v27220	all isoforms
		VDRC	46687	w^{1118}; P{GD16420}v46687	all isoforms
		VDRC	6873	w^{1118}; P{GD3148}v6873/TM3	all isoforms
put	*Acvr2a*	BDSC	27514	y^1 v^1; P{TRiP.JF02664}attP2	all isoforms
		BDSC	39025	y^1 sc* v^1; P{TRiP.HMS01944}attP40	all isoforms
		VDRC	107071	P{KK102676}VIE-260B	all isoforms
robo1	*Robo2*	BDSC	31287	y^1 v^1; P{TRiP.JF01230}attP2	all isoforms
		BDSC	35768	y^1 sc* v^1; P{TRiP.HMS01517}attP2	all isoforms
		BDSC	31663	y^1 v^1; P{TRiP.JF01456}attP2	all isoforms
		BDSC	39027	y^1 sc* v^1; P{TRiP.HMS01946}attP40	all isoforms
		VDRC	100624	P{KK108817}VIE-260B	all isoforms
Sema1a	*Sema6-A/D*	BDSC	29554	y^1 v^1; P{TRiP.JF03231}attP2	all isoforms
		BDSC	34320	y^1 sc* v^1; P{TRiP.HMS01307}attP2	all isoforms

		VDRC	104505	P{KK109430}VIE-260B	all isoforms
Sema1b	*Sema6B*	BDSC	28588	y^1 v^1; P{TRiP.HM05076}attP2	all isoforms
		VDRC	107233	P{KK104666}VIE-260B	all isoforms
Sema2a	*Sema3A*	BDSC	29519	y^1 v^1; P{TRiP.HM05196}attP2	all isoforms
		VDRC	15810	w^{1118}; P{GD5476}v15810/TM3	all isoforms
		VDRC	15811	w^{1118}; P{GD5476}v15811/CyO	all isoforms
Sema5c	*Sema5-A/B*	BDSC	29436	y^1 v^1; P{TRiP.JF03372}attP2	all isoforms
		VDRC	1052	w^{1118} P{GD5}v1052	all isoforms
shot	*Dst*	BDSC	28336	y^1 v^1; P{TRiP.JF02971}attP2	all isoforms
sli	*Slit3*	BDSC	31467	y^1 v^1; P{TRiP.JF01228}attP2	all isoforms
		BDSC	31468	y^1 v^1; P{TRiP.JF01229}attP2	all isoforms
sm	*Hnrnpl*	VDRC	108351	P{KK108588}VIE-260B	all isoforms
stan	*Celsr-1/3*	BDSC	35050	y^1 sc^* v^1; P{TRiP.HMS01464}attP2	2 out of 7 isoforms (stan-RA, RB)
		BDSC	26022	y^1 v^1; P{TRiP.JF02047}attP2	all isoforms
		VDRC	107993	P{KK100512}VIE-260B	all isoforms
Sdc	*Sdc-1/2*	BDSC	51723	y^1 sc* v^1; P{TRiP.HMC03265}attP2	all isoforms
		VDRC	13322	w^{1118}; P{GD4545}v13322	all isoforms
tok	*Bmp1*	VDRC	2656	w^{1118}; P{GD245}v2656	all isoforms
tutl	*Igsf9*	BDSC	54850	y^1 v^1; P{TRiP.HMJ21587}attP40	all isoforms
		VDRC	108746	P{KK108880}VIE-260B	all isoforms
unc-5	*Unc5c*	BDSC	33756	y^1 sc^* v^1; P{TRiP.HMS01099}attP2	all isoforms
		VDRC	110155	P{KK102074}VIE-260B	all isoforms
uzip	-	BDSC	29558	y^1 v^1; P{TRiP.JF03237}attP2	all isoforms
wit	*Bmpr2*	BDSC	41906	y^1 sc^* v^1; P{TRiP.HMS02298}attP2	all isoforms
		BDSC	25949	y^1 v^1; P{TRiP.JF01969}attP2	all isoforms
		VDRC	103808	P{KK100911}VIE-260B	all isoforms
Wnk	*Wnk*	BDSC	42521	y^1 v^1; P{TRiP.HMJ02087}attP40	all isoforms
		VDRC	106928	P{KK102654}VIE-260B	all isoforms
β-Spec	*Sptbn1*	BDSC	38533	y^1 sc* v^1; P{TRiP.HMS01746}attP40	all isoforms

Supplementary Table S7. List of control lines used in the study

attP2 insertion TRiP line injection strain	BDSC	36303	y^1 v^1; P{CaryP}attP2
attP40 insertion TRiP line injection strain	BDSC	36304	y^1 v^1; P{CaryP}attP40
KK line injection strain	VDRC	60100	y,w^{1118}; P{attP,y^+,w[3`]}
GD line injection strain	VDRC	60000	w^{1118}
UAS-dsRNA-GFP on 2nd chromosome	BDSC	9331	w^{1118}; P{UAS-GFP.dsRNA.R}143
UAS-dsRNA-GFP on 3rd chromosome	BDSC	9330	w^{1118}; P{UAS-GFP.dsRNA.R}142
UAS-dsRNA-mCherry (additional control for VALIUM 20 attP2 insertion lines)	BDSC	35785	y1 sc* v1; P{VALIUM20-mCherry}attP2
UAS-mCherry (additional control for VALIUM 10 and VALIUM 20 lines)	BDSC	35787	y1 sc* v1; P{VALIUM10-mCherry}attP2
UAS-dsRNA-lacZ (additional control for KK lines)	VDRC	51446	w1118; P{GD936}v51446

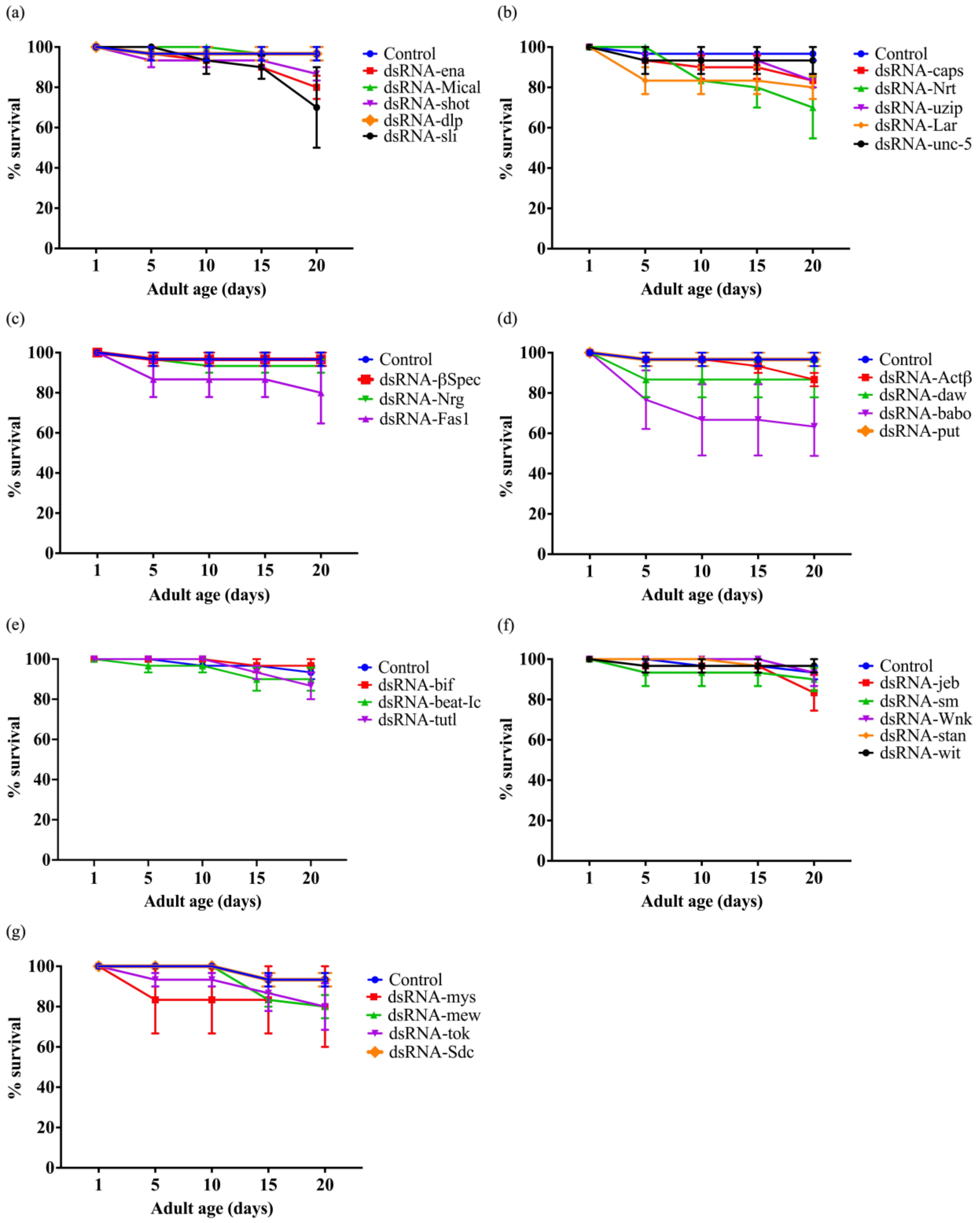

Supplementary Figure S1. Survival analysis curves of axon guidance genes that did not show a significant effect in the GeneSwitch screen. Data shown are mean ± SEM ($p<0.05$, two-way ANOVA and Tukey's multiple comparison tests; $n=30$).

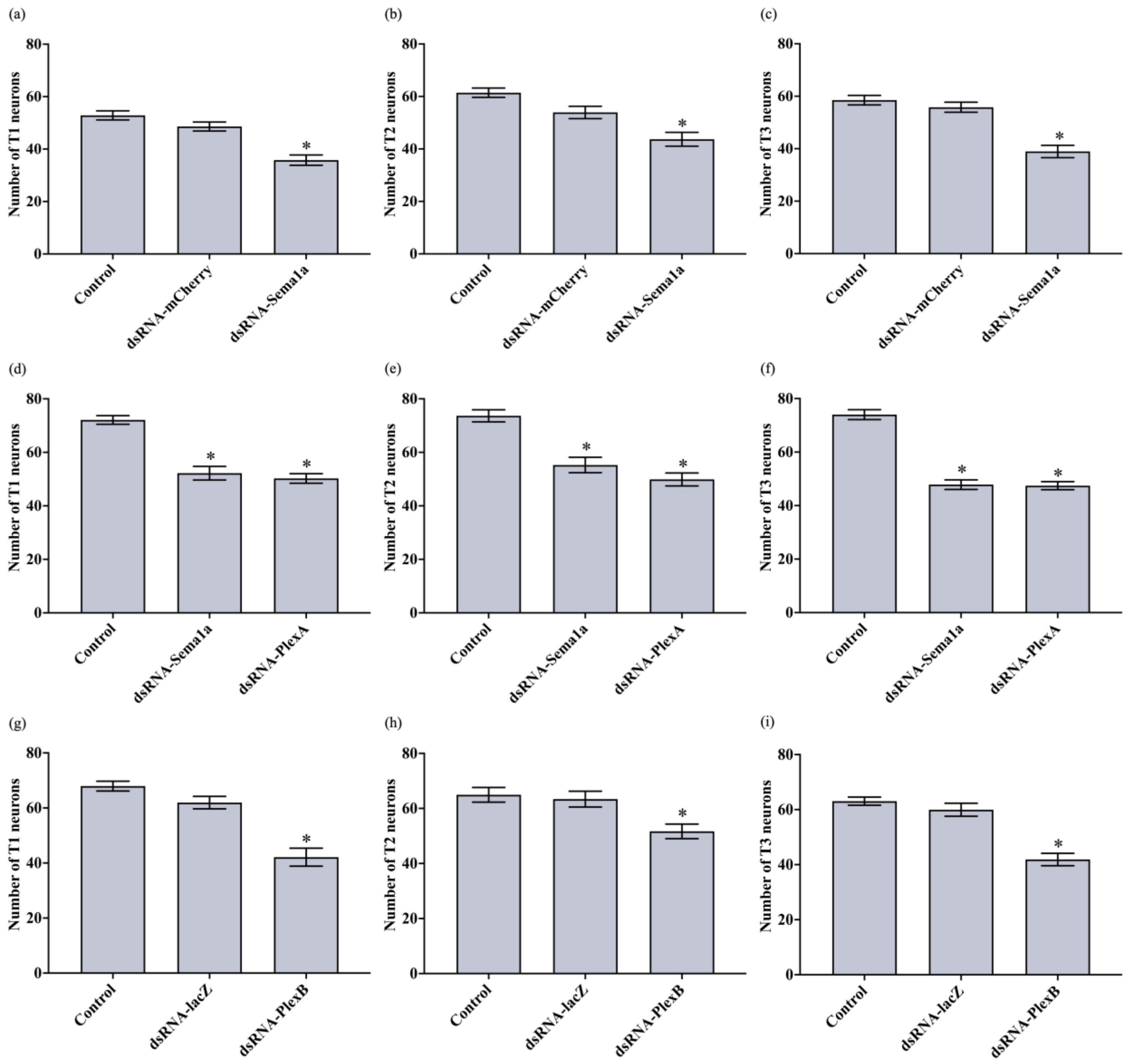

Supplementary Figure S2. Knockdown of Semaphorins and Plexins using additional *UAS-dsRNA* lines corroborates the motor neuron death phenotype. Quantification of adult leg motor neurons in T1 (a, d, g), T2 (b, e, h) and T3 (c, f, i) thoracic segments, following 9 days of axon guidance gene knockdown using additional *UAS-dsRNA* lines against Semaphorins and Plexins. Data shown are mean ± SEM ('*' denotes a significant difference from the respective control(s); $p < 0.05$, one-way ANOVA and Tukey's multiple comparison tests; n=20).
